# Comparison of the Humphrey Field Analyzer and Photopic Negative Response of Focal Macular Electroretinograms in the Evaluation of the Relationship Between Macula Structure and Function

**DOI:** 10.3389/fmed.2021.649971

**Published:** 2021-02-26

**Authors:** Kazuyuki Hirooka, Kenji Yokoyama, Kana Tokumo, Yoshiaki Kiuchi

**Affiliations:** Department of Ophthalmology and Visual Science, Graduate School of Biomedical Sciences, Hiroshima University, Hiroshima, Japan

**Keywords:** visual field, optical coherance tomography, electroretinogram (ERG), glaucoma, structure-function

## Abstract

**Purpose:** To investigate the association between macular inner retinal layer thickness and macula visual field (VF) mean deviation as measured by the Humphrey Field Analyzer (HFA) or macular function as measured by focal macular electroretinograms (ERGs) in patients with glaucoma.

**Methods:** The participants in this cross-sectional study were 71 patients with glaucoma and 10 healthy controls. Macular inner retinal layer thickness and function were measured in all participants using optical coherence tomography (OCT) and HFA or focal macular ERGs, respectively. Macular OCT images were segmented into the macular retinal nerve fiber layer (mRNFL), macular ganglion cell layer/inner plexiform layer (GCL/IPL), and ganglion cell complex (GCC). Spearman correlation analysis was used to assess the relationship between macular inner retinal layer thickness and function.

**Results:** Focal macular ERGs were composed of a negative wave (N1), a positive wave (P1), and a slow negative wave (N2). The N2 response density was significantly reduced in eyes with glaucoma, and was significantly associated with the thickness of the mRNFL (*R* = 0.317), GCL/IPL (*R* = 0.372), or GCC (*R* = 0.367). The observed structure–function relationship was also significantly correlated with the HFA VF mean deviation for each thickness [mRNFL (*R* = 0.728), GCL/IPL (*R* = 0.603), or GCC (*R* = 0.754)].

**Conclusions:** Although a significant correlation was found between the N2 response density and the thickness of the macular inner layer, the observed structure–function relationship with the mean deviation of the HFA VF was higher than that of the N2 response density.

## Introduction

Glaucoma is a group of ocular diseases known to be characterized by retinal ganglion cell (RGC) soma and axon loss (1, 2). As about 50% of the RGCs are within 4.5 mm of the foveal center (3), measuring macular RGC function could be useful for diagnosing glaucoma or predicting disease progression. Some studies investigated the relationship between local sensitivity loss on 10-2 visual field (VF) loss and macular ganglion cell/inner plexiform layer (GCL/IPL) thickness (4–7). Clarifying the relationship between macular GCL/IPL thickness and central visual function could help clinicians gain a better understanding of how to detect glaucomatous damage at the early stage and disease progression. The Humphrey Field Analyzer (HFA; Carl Zeiss Meditec, Dublin, CA) has been confirmed to have high test–retest variability, with fixation errors being one of the major factors (8).

The photopic negative response (PhNR), which originates from the activity of RGCs and their axons (9), is a negative wave that follows the photopic b-wave. Increasing evidence has shown that the PhNR can be useful in evaluating the functional condition of neurons in patients with glaucoma (10, 11). The amplitude of the focal PhNR has been shown to be significantly correlated with a reduction in both visual sensitivity as determined by standard automated perimetry (SAP) (11) and retinal nerve fiber layer (RNFL) thickness (10) in patients with glaucoma. The PhNR recorded from the macular area can be used to assess the function of associated RGCs (12). The PhNR recorded using multifocal electroretinograms (mfERGs) with pseudorandom sequence stimulation has been found to be reduced in patients with glaucoma compared with controls, and this reduction in multifocal PhNR (mfPhNR) amplitude was correlated with disease severity (13). Due to recent improvements in the mfERG technique, the pupil does not need to be dilated before recording the mfPhNR.

To improve the ability to detect the presence and progression of glaucomatous damage, numerous studies have applied spectral-domain optical coherence tomography (OCT) to examine the association between structural and functional damage. Given this background, the present study aimed to compare macular function measurements made by the HFA and focal macular PhNR, and to assess whether any potential relationships exist between these measurements and the thickness of the macular inner retinal layer.

## Materials and Methods

### Patients

This cross-sectional study was carried out at Hiroshima University Hospital. The participants were all patients examined between November 2019 and August 2020. Before the study began, in accordance with the principles outlined in the Declaration of Helsinki, all participants were given a detailed explanation of the study purpose and methods and then asked to provide written informed consent. This study was approved by the Institutional Review Board of the Hiroshima University Faculty of Medicine.

First, all participants underwent a complete ophthalmic examination, which included visual acuity testing with refraction, intraocular pressure, gonioscopy, and a dilated fundus examination with stereoscopic biomicroscopy of the optic nerve head using indirect ophthalmoscopy and a slit lamp. Participants with a best-corrected visual acuity of ≥20/25, a spherical error within a range of +4.0 and −6.0 diopters, a cylinder within ± 2.0 diopters, an axial length <26 mm, and open angles (grades 3 and 4 according to the Shaffer grading system) were included in the analysis. An optical biometer (IOLMaster, Carl Zeiss Meditec) was used to acquire axial length. Participants with a history of retinal pathology or neurologic disease or who had undergone a retinal laser procedure or either retinal or intraocular surgery were excluded. If both eyes met the inclusion criteria, the right eye was assessed. Control subjects were required to have an intraocular pressure ≤ 21 mmHg and a normal VF. All included eyes had to show the following structural glaucomatous changes to meet the definition of glaucoma: a vertical cup-disc asymmetry of ≥0.2 between the eyes, a cup-to-disc ratio of ≥0.6, neuroretinal rim narrowing, notches, localized pallor, or RNFL defects with glaucomatous VF loss in the corresponding hemifield. To meet the definition of glaucomatous VF, the participant had to have undergone a glaucoma hemifield test outside of the normal limits in a minimum of two consecutive baseline tests, with at least three contiguous test points within the same hemifield on a pattern deviation plot at *P* <1% and at least one contiguous test point at *P* < 0.5%, after excluding test points that were on the edge of the field or directly above and below the blind spot.

### Measurement of Macular Inner Retinal Layer Thickness

Raster scanning [scan density of 512 (vertical) × 128 (horizontal) scans] of a 7 mm^2^ area centered on the fovea was performed using a high-resolution fundus camera (Topcon 3D OCT-2000; Topcon, Inc., Tokyo, Japan). The built-in protocol measured a 6 × 6-mm area centered on the fovea using embedded software. The data were divided into 10 × 10 grids and exported by the Topcon software. Then, the mean thickness of the macular retinal nerve fiber layer (mRNFL), GCL/IPL, and ganglion cell complex (GCC), which consists of the mRNFL and GCL/IPL, were calculated. Images with a quality factor <30 were excluded from the analysis.

### Visual Sensitivity of the 10-2 HFA

Visual sensitivity was examined using static automated white-on-white threshold perimetry (HFA; 10-2 Swedish Interactive Threshold Algorithm Standard test). The VF results were considered reliable when the fixation losses and false-positive/false-negative rates were <20%. The subsequent analyses use only reliable test data.

### mfERG Recordings

**Figure 1:**
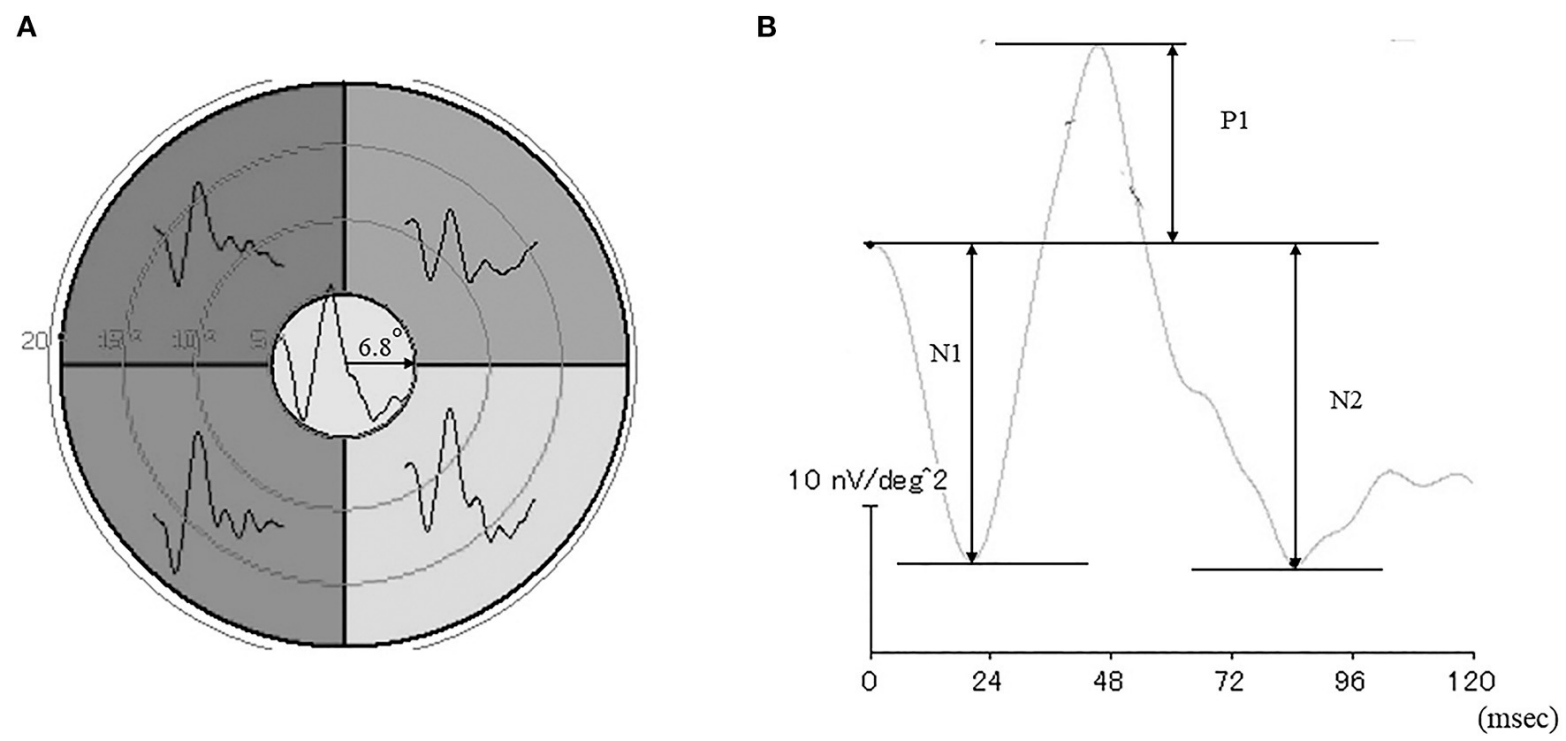
Stimulus patterns used to elicit the multifocal electroretinograms (mfERGs) **(A)**. Representative waveforms of the mfERGs recorded from five retinal loci. **(B)** Focal electroretinogram.

As described in a previous study on mfERG recordings (13) and shown in Figure 1A, stimuli consisting of five stimulus elements were generated on a cathode-ray tube monitor (VERIS? 7, Electro-Diagnostic Imaging, San Mateo, CA). mfERGs were elicited by a circular stimulus with a 6.8° radius centered on the fovea and a quarter of an annulus placed around the macula, with the radius of the outer border of the annulus set to 20°. White (200 cd/mm^2^) or black (4 cd/mm^2^) elements were presented in a pseudorandom binary m-sequence at a frequency of 6.25 Hz, with a steady background of 100 cd/m^2^ surrounding the stimulus field.

A Burian-Allen bipolar contact lens electrode (Hansen Ophthalmic Laboratories, Coralville, IA) was placed on the cornea following corneal anesthesia. A chloride silver electrode as the ground electrode was placed on the left ear lobe. All responses were digitally band-pass filtered between 3 and 30 Hz. VERIS software (VERIS Science 4.1.1; Maya, Nagoya, Japan) was used to analyzed the mfERGs. The local retinal responses from the five different retinal loci were averaged to obtain the all-trace waveforms of the first-order kernels (Figure 1A) The response density of focal ERGs in the center area were evaluated as a macular function.

The N1 and P1 amplitudes were measured from the baseline to the trough of the first negative response and the peak of the following positive wave, respectively, and the N2 amplitude was measured from the baseline to the following trough (Figure 1B). The focal ERG amplitudes were expressed as response density (nV/deg^2^), which represents the amplitude as a function of the stimulus area.

### Statistical Analysis

For continuous variables, variance equality was assessed using Levene's test. Based on the results obtained, a Student's *t*-test or Welch's test was used to assess differences between the control and glaucoma groups. The chi-square test for categorical parameters was used to assess differences between the control and glaucoma groups. Spearman rank order correlations were used to examine the correlation between mRNFL, GCL/IPL, and GCC thickness and VF mean deviation or N2 response density, and tests of equality of dependent correlation coefficients were used to evaluate comparisons of the strength of the structure–function association. All statistical values are presented as means ± standard deviations (SDs), with *P* <0.05 considered to be statistically significant. JMP software (version 15; SAS Inc., Cary, NC) was used for all statistical analyses.

## Results

### Demographic Characteristics

The demographic characteristics of the 71 patients with glaucoma and 10 healthy controls who participated in the study are shown in Table 1. Disease grade in the glaucomatous eyes of the 71 patients, which was based on the standard VF severity grading scale (14), ranged from early to moderate, with 13 (18.3%), 17 (23.9%), and 41 (57.7%) eyes classified as early, moderate, and severe, respectively.

**Table 1:** Clinical characteristics of the study population.

	**Glaucoma (*n* = 71)**	**Normal (*n* = 10)**	***P-*value**
Age (y)	71.0 ± 1.5	68.2 ± 3.9	0.50
Gender (M/F)	39/32	6/4	0.76
Diagnosis			
POAG	46		
NTG	8		
EG	17		
Refraction (D)	−1.3 ± 0.2	−0.3 ± 0.6	0.13
Axial length (mm)	24.0 ± 0.1	23.5 ± 0.3	0.046

*M, male; F, female; POAG, primary open-angle glaucoma; NTG, normal-tension glaucoma; EG, exfoliation glaucoma; D, diopter*.

### Comparison of the Normal and Glaucoma Groups

A significant difference in mRNFL, GCL/IPL, or GCC thickness was observed between the glaucoma and healthy control groups, as shown in Table 2. The mean deviation was significantly lower in the glaucomatous than in the healthy eyes, and the N2 response density was significantly reduced in the glaucomatous eyes, as shown in Table 2.

**Table 2:** Macular inner retinal layer thickness and function.

	**Glaucoma**	**Normal**	***P*-value**
GCC thickness (μm)	71.7 ± 1.2	101.0 ± 3.2	<0.001^a^
GCL/IPL thickness (μm)	51.6 ± 0.6	63.4 ± 1.5	<0.001^a^
mRNFL thickness (μm)	20.2 ± 0.9	37.8 ± 2.3	<0.001^b^
MD of 10-2 (dB)	14.01 ± 0.87	1.06 ± 2.31	<0.001^b^
N2 response density (nV/deg^2^)	7.52 ± 0.76	14.14 ± 2.04	0.003^a^

*GCC, ganglion cell complex; GCL, ganglion cell layer; IPL, inner plexiforma layer; mRNFL, macular retinal nerve fiber layer; MD, mean deviation*.

a *Student's t-test*.

b *Welch test*.

### Correlation Between Macular Inner Layer Thickness and Mean Sensitivity and N2 Response Density

**Figure 2:**
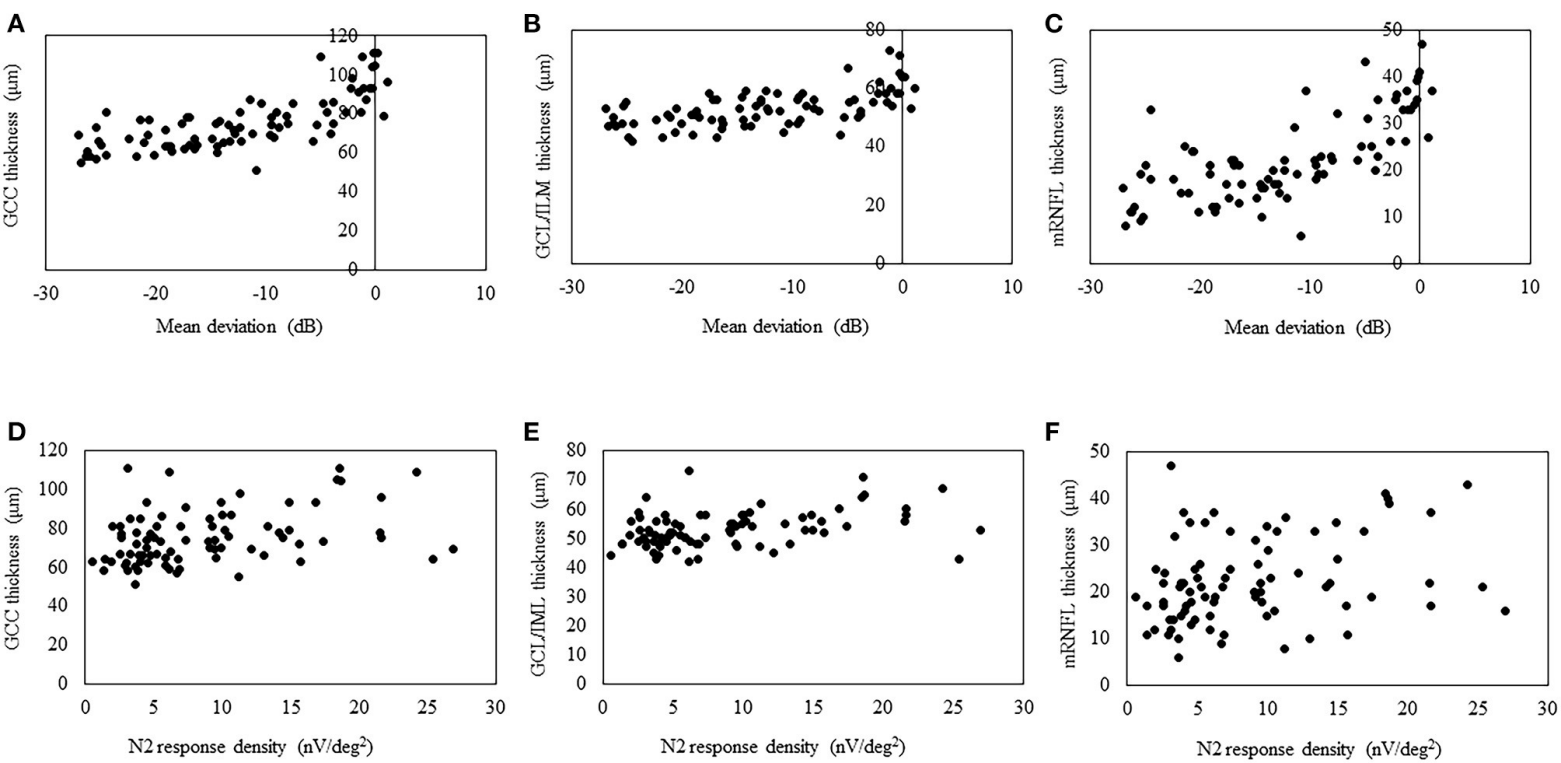
Scatterplots showing the association between the 3D OCT-2000 thickness parameters and the corresponding retinal sensitivity (decibels) measured by the Humphrey Field Analyzer or center N2 response density. Association between the average thickness of the ganglion cell complex (GCC) **(A)**, macular ganglion cell layer/inner plexiform layer (GCL/IPL) **(B)**, or macular retinal nerve fiber layer (mRNFL) **(C)** and macular mean deviation. Association between the average thickness of the GCC **(D)**, GCL/IPL **(E)**, or mRNFL **(F)** and the center N2 response.

The structure–function relationship was evaluated based on the mRNFL, GCL/IPL, or GCC thickness and VF mean deviation or N2 response density (Figure 2 and Table 3). In each mRNFL, GCL/IPL, or GCC thickness, the structure–function relationship observed HFA VF mean deviation was higher than those of N2 response density. The Spearman correlation coefficient was the highest (0.754) for the GCC thickness-HFA VF mean sensitivity measurements. Table 4 shows the structure–function relationship in each glaucoma type.

**Table 3:** Comparison of the strength of the structure-function relationship between the Humphrey Field Analyzer and focal electroretinograms.

	**Statistic**	**HFA**	**Focal ERG**	***P*-value**
GCC	Correlation coefficient	0.754	0.367	<0.001
	95% bootstrapped CI	0.625–0.826	0.172–0.550	
GCL/IPL	Correlation coefficient	0.603	0.372	0.06
	95% bootstrapped CI	0.444–0.727	0.155–0.537	
mRNFL	Correlation coefficient	0.728	0.317	<0.001
	95% bootstrapped CI	0.599–0.813	0.089–0.488	

*HFA, humphrey field analyzer; ERG, electroretinogram; CI, confidence interval; GCC, ganglion cell complex; GCL, ganglion cell layer; IPL, inner plexiform layer; mRNFL, macular retinal nerve fiber layer*.

**Table 4:** Comparison of the strength of structure-function relationship between Humphrey Field Analyzer and multifocal electroretinogram in each glaucoma type.

		**POAG (*****n*** **=** **46)**		**NTG (*****n*** **=** **8)**		**EG (*****n*** **=** **17)**	
	**Statistic**	**HFA**	**Focal ERG**	**HFA**	**Focal ERG**	**HFA**	**Focal ERG**
GCC	Correlation coefficient	0.783	0.367	0.715	0.336	0.676	0.486
	95% bootstrapped CI	0.661–0.864	0.087–0.538	0.270–0.908	0.263–0.749	0.405–0.838	0.138–0.727
GCL/IPL	Correlation coefficient	0.614	0.313	0.514	0.319	0.615	0.491
	95% bootstrapped CI	0.429–0.750	0.067–0.524	0.051–0.830	0.281–0.740	0.314–0.803	0.144–0.703
mRNFL	Correlation coefficient	0.782	0.299	0.799	0.347	0.659	0.457
	95% bootstrapped CI	0.661–0.864	0.052–0.513	0.444–0.938	0.252–0.754	0.380–0.829	0.102–0.709

*POAG, primary open-angle glaucoma; NTG, normal-tension glaucoma; EG, exfoliation glaucoma; HFA, humphrey field analyzer; ERG, electroretinogram; CI, confidence interval; GCC, ganglion cell complex; GCL, ganglion cell layer; IPL, inner plexiform layer; mRNFL, macular retinal nerve fiber layer*.

## Discussion

The retina contains about 1.07 million RGCs on average, approximately half of which are located within 4.5 mm of the foveal center (3, 15). During the early stages of glaucoma, RGC loss is evident around the fovea (16), which highlights the importance of assessing the central macular structure–function relationship. The N2 component of mfERGs recorded from the central area represents the RGC activity in the corresponding macular area (13).

### Structure–Function Relationship of Glaucomatous Damage

Some researchers have recently reported on the diagnostic performance of mfERG in glaucoma patients (17, 18) or an animal model of glaucoma (19). The combination of mfERG and OCT improved diagnostic performance and monitoring of disease progression (17). By analyzing the mfPhNR/b-wave ratio, Al-Nosairy et al. (18) achieved the best performance for discriminating between controls and glaucoma suspects. The diagnostic performance and structure–function relationship were strongest for mfERG when compared with full-field flash ERG PhNR or pattern-reversal ERG in an experimental animal model of glaucoma (19). A combined approach using structural and functional assessment of glaucomatous retinal damage offers great promise for uncovering the interrelationship between the different components of ocular damage in glaucoma.

### Correlation Between Macular Inner Layer Thickness and N2 Response Density

The high test–retest variability of SAP is often explained by poor patient vigilance and inattention in subjective examinations. By contrast, measurements of mfPhNR amplitude tend to show better test–retest reliability because of this is an objective test (20). Therefore, we hypothesized that the structure–function relationship for the observed N2 response would be higher than that for the HFA VA mean deviation. Although a significant correlation was found between N2 response density and macular inner layer thickness in this study, the structure–function relationship for the observed HFA VF mean deviation was higher than that for the N2 response density. Macular focal ERGs were elicited by a circular stimulus with a 6.8° radius centered on the fovea. The built-in protocol measured a 6 × 6-mm area centered on the fovea corresponding to a 20° square of the retina in the macular area. The 3D-OCT used in this study and the 10-2 HFA measure similar macular areas (the 10-2 HFA analyzes 68 data points located within a central arc of 10°). Therefore, we assume that the results may be affected by the measurement area of each instrument. Moreover, the N2 may not represent the neural activity of RGCs only. In rodents, the PhNR has been shown to be affected by the neural activity of amacrine cells (21, 22).

In the present study, although a significant correlation was observed between the N2 response density and GCC thickness, the correlation coefficient was lower than that in a previous study (*R* = 0.363 vs. 0.575, respectively) (13), in which SD-OCT (RS-3000 Advance; Nidek Co., Ltd.) was used to obtain the GCC thickness. It is therefore difficult to compare the strength of the structure–function relationship in this study with that in their study because it can be affected by sample size, disease severity, and OCT instruments.

### Limitations

This study did have some limitations. First, the present study did not include any patients with preperimetric glaucoma; such patients should be examined and compared with regard to the structure–function relationship in a future study. Second, although we observed no obvious differences in the structure–function relationship among patients with primary open-angle, normal tension, or exfoliation glaucoma, the sample size was small. Therefore, a large number of subjects will need to be closely examined for each glaucoma type in a future study.

## Conclusions

The results of this study revealed that the N2 response density was affected by glaucoma in the central macular area. In addition, a significant correlation was found between the N2 amplitude and macular inner layer thickness; however, this correlation was weaker than that between the macular inner layer thickness and HFA VF mean deviation.

## Data Availability Statement

The datasets presented in this study can be found in online repositories. The names of the repository/repositories and accession number(s) can be found in the article/supplementary material.

## Ethics Statement

The studies involving human participants were reviewed and approved by Institutional Review Board of the Hiroshima University Faculty of Medicine. The patients/participants provided their written informed consent to participate in this study.

## Author Contributions

KH: conception of design of the work acquisition, analysis, and interpretation of the data final approval of the version to be published and agreement to be accountable for all aspects of the work in ensuring that questions related to the accuracy and integrity of any part of the work are appropriately investigated and resolved. KY: acquisition of the data final approval of the version to be published and agreement to be accountable for all aspects of the work in ensuring that questions related to the accuracy and integrity of any part of the work are appropriately investigated and resolved. KT: acquisition of the data final approval of the version to be published and agreement to be accountable for all aspects of the work in ensuring that questions related to the accuracy and integrity of any part of the work are appropriately investigated and resolved. YK: final approval of the version to be published and agreement to be accountable for all aspects of the work in ensuring that questions related to the accuracy and integrity of any part of the work are appropriately investigated and resolved. All authors contributed to the article and approved the submitted version.

## Conflict of Interest

The authors declare that the research was conducted in the absence of any commercial or financial relationships that could be construed as a potential conflict of interest.
